# Supplementary material for: Molecular species identification of bushmeat recovered from the Serengeti ecosystem in Tanzania
Source: PLoS One. 2020 Sep 14;15(9):e0237590. doi: 10.1371/journal.pone.0237590 (PMC7489505; doi:10.1371/journal.pone.0237590)
Supplement: S1 Table — This table corresponds to the data and proportions in Fig 2. (DOCX) [file pone.0237590.s001.docx]

**S1 Table. Pairwise comparison of misreporting percentages between the top 5 most abundant species.** This table corresponds to the data and proportions in Figure 2.
