# Supplementary material for: Molecular species identification of bushmeat recovered from the Serengeti ecosystem in Tanzania
Source: PLoS One. 2020 Sep 14;15(9):e0237590. doi: 10.1371/journal.pone.0237590 (PMC7489505; doi:10.1371/journal.pone.0237590)
Supplement: S2 Table — Proportions were compared using a two-tailed Z Score analysis at a significance level of 0.05. (DOCX) [file pone.0237590.s002.docx]

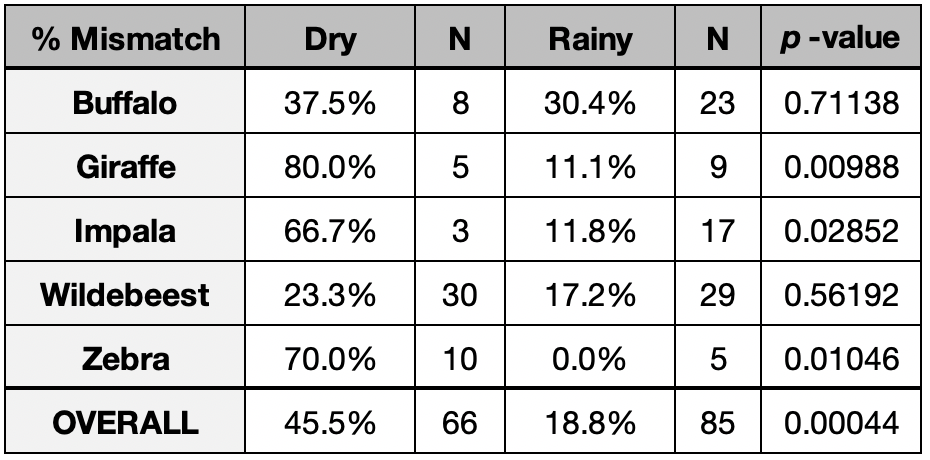


**S2 Table. Proportion of mismatched samples during the dry and rainy seasons**. Proportions were compared using a two-tailed Z Score analysis at a significance level of 0.05.
