# Supplementary material for: Molecular species identification of bushmeat recovered from the Serengeti ecosystem in Tanzania
Source: PLoS One. 2020 Sep 14;15(9):e0237590. doi: 10.1371/journal.pone.0237590 (PMC7489505; doi:10.1371/journal.pone.0237590)
Supplement: Attachment — Submitted filename: Response to Reviewers 2.docx [file pone.0237590.s003.docx]

PONE-D-20-05756R1
Molecular species identification of bushmeat recovered from the Serengeti ecosystem in Tanzania
Dr Megan Ann Schilling

Dear Dr Schilling,

Thank you for submitting your manuscript entitled "Molecular species identification of bushmeat recovered from the Serengeti ecosystem in Tanzania" to PLOS ONE. Your manuscript files have been checked in-house but before we can proceed we need you to address the following issues:

1. Your ethics statement must appear in the Methods section of your manuscript. If your ethics statement is written in any section besides the Methods, please move it to the Methods section and delete it from any other section. Please also ensure that your ethics statement is included in your manuscript, as the ethics section of your online submission will not be published alongside your manuscript.

2. To comply with PLOS ONE submissions requirements for field studies, please provide the following information in the Methods section of the manuscript and in the “Ethics Statement” field of the submission form (via “Edit Submission”):
a) Provide the name of the authority who issued the permission for each location (for example, the authority responsible for a national park or other protected area of land or sea, the relevant regulatory body concerned with protection of wildlife, etc.). If the study was carried out on private land, please confirm that the owner of the land gave permission to conduct the study on this site.
b) For any locations/activities for which specific permission was not required, please
- i. state clearly that no specific permissions were required for these locations/activities, and provide details on why this is the case
- ii. confirm that the field studies did not involve endangered or protected species

For more information about PLOS ONE submissions requirements for field studies, please refer to <http://journals.plos.org/plosone/s/submission-guidelines#loc-animal-research>.

Response: The methods section of the manuscript was updated to include “Permissions and permits to collect samples in the Serengeti National Park was granted through the Tanzania Commission for Science and Technology, Tanzania Wildlife Research Institute (permit number TWRI/RS-331/VOL.II/2013/58 and TWRI/RS-331/VOL.II/2013/88), Tanzania National Parks (permit number TNP/HQ/C.10/1), Ngorongoro Conservation Area Authority (permit number NCAA/D/240/Vol.XXVIII/54), and Tanzania Wildlife Authority (permit number CB.517/519/01/14).”

3. We note that Figure 1 in your submission contain [map/satellite] images which may be copyrighted. All PLOS content is published under the Creative Commons Attribution License (CC BY 4.0), which means that the manuscript, images, and Supporting Information files will be freely available online, and any third party is permitted to access, download, copy, distribute, and use these materials in any way, even commercially, with proper attribution. For these reasons, we cannot publish previously copyrighted maps or satellite images created using proprietary data, such as Google software (Google Maps, Street View, and Earth). For more information, see our copyright guidelines: <http://journals.plos.org/plosone/s/licenses-and-copyright>.

Response: The authors generated the map in Figure 1. This map was not a copyrighted image from any other source.

We require you to either (1) present written permission from the copyright holder to publish these figures specifically under the CC BY 4.0 license, or (2) remove the figures from your submission:

1. You may seek permission from the original copyright holder of Figure(s) [#] to publish the content specifically under the CC BY 4.0 license.

We recommend that you contact the original copyright holder with the Content Permission Form (http://journals.plos.org/plosone/s/file?id=7c09/content-permission-form.pdf) and the following text:
“I request permission for the open-access journal PLOS ONE to publish XXX under the Creative Commons Attribution License (CCAL) CC BY 4.0 (http://creativecommons.org/licenses/by/4.0/). Please be aware that this license allows unrestricted use and distribution, even commercially, by third parties. Please reply and provide explicit written permission to publish XXX under a CC BY license and complete the attached form.”

Please upload the completed Content Permission Form or other proof of granted permissions as an "Other" file with your submission.

In the figure caption of the copyrighted figure, please include the following text: “Reprinted from [ref] under a CC BY license, with permission from [name of publisher], original copyright [original copyright year].”

2. If you are unable to obtain permission from the original copyright holder to publish these figures under the CC BY 4.0 license or if the copyright holder’s requirements are incompatible with the CC BY 4.0 license, please either i) remove the figure or ii) supply a replacement figure that complies with the CC BY 4.0 license. Please check copyright information on all replacement figures and update the figure caption with source information. If applicable, please specify in the figure caption text when a figure is similar but not identical to the original image and is therefore for illustrative purposes only.
The following resources for replacing copyrighted map figures may be helpful:

USGS National Map Viewer (public domain): http://viewer.nationalmap.gov/viewer/
The Gateway to Astronaut Photography of Earth (public domain): http://eol.jsc.nasa.gov/sseop/clickmap/
Maps at the CIA (public domain): https://www.cia.gov/library/publications/the-world-factbook/index.html and https://www.cia.gov/library/publications/cia-maps-publications/index.html
NASA Earth Observatory (public domain): http://earthobservatory.nasa.gov/
Landsat: http://landsat.visibleearth.nasa.gov/
USGS EROS (Earth Resources Observatory and Science (EROS) Center) (public domain): http://eros.usgs.gov/#
Natural Earth (public domain): http://www.naturalearthdata.com/

Your manuscript has been returned to your account. Please log on to PLOS Editorial Manager at https://www.editorialmanager.com/pone/ to access your manuscript.

Your manuscript can be found in the "Revisions Sent Back to the Author" link under the New Submissions menu. After you have made the changes requested above, please be sure to view and approve the revised PDF after rebuilding the PDF to complete the resubmission process.

Please note that these changes have been requested to comply with submission guidelines and your manuscript will *not* be sent to review until you have fully adhered to our requests. Once your paper has been seen by an Editor we may return it to you for further information or amendments.

We ask that you address this request within 28 days. If you require additional time, please email the journal office. We are happy to grant extensions of up to one month past this due date. If we have not heard from you within 28 days, your manuscript will be withdrawn from Editorial Manager.

Thank you for submitting your work to PLOS ONE.

Kind regards,

Zsoka Murakozi
PLOS ONE
